# Experimental and Theoretical Study of the Electronic Structures of Lanthanide Indium Perovskites LnInO_3_

**DOI:** 10.1021/acs.jpcc.0c11592

**Published:** 2021-03-11

**Authors:** P. Hartley, R. G. Egdell, K. H. L. Zhang, M. V. Hohmann, L. F. J. Piper, D. J. Morgan, D. O. Scanlon, B. A. D. Williamson, A. Regoutz

**Affiliations:** † Department of Chemistry, Inorganic Chemistry Laboratory, University of Oxford, South Parks Road, Oxford OX1 3QR, U.K.; ‡ State Key Laboratory of Physical Chemistry of Solid Surfaces, College of Chemistry and Chemical Engineering, Xiamen University, Xiamen 361005, People’s Republic of China; § Institute of Materials Science, Surface Science Division, Technische Universität Darmstadt, Darmstadt 64287, Germany; ∥ WMG, The University of Warwick, Coventry CV4 7AL, U.K.; ⊥ Department of Applied Physics & Astronomy, Binghamton University, State University of New York, Binghamton, New York 13902, United States; # Cardiff Catalysis Institute, School of Chemistry, Cardiff University, Park Place, Cardiff CF10 3AT, U.K.; ¶ Department of Chemistry, University College London, 20 Gordon Street, London WC1H 0AJ, U.K.; ∇ Thomas Young Centre, University College London, Gower Street, London WC1E 6BT, U.K.; ○ Diamond Light Source Ltd., Diamond House, Harwell Science and Innovation Campus, Didcot, Oxfordshire OX11 0DE, U.K.; ⧫ Department of Materials Science and Engineering, Norwegian University of Science and Technology (NTNU), Trondheim 7491, Norway

## Abstract

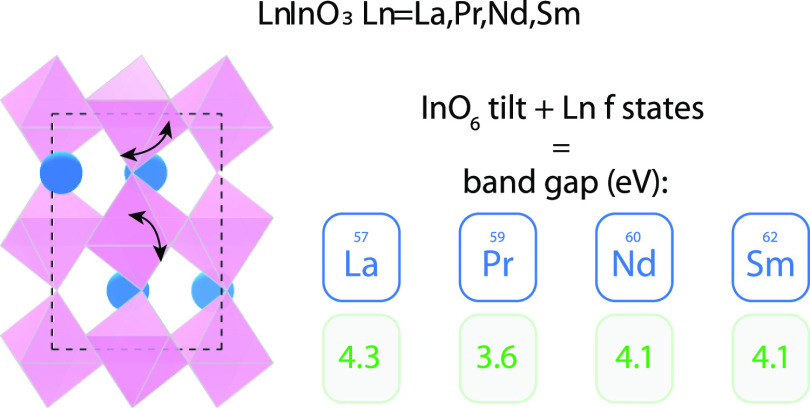

Ternary lanthanide indium oxides LnInO_3_ (Ln = La, Pr, Nd, Sm) were synthesized by high-temperature solid-state reaction and characterized by X-ray powder diffraction. Rietveld refinement of the powder patterns showed the LnInO_3_ materials to be orthorhombic perovskites belonging to the space group *Pnma*, based on almost-regular InO_6_ octahedra and highly distorted LnO_12_ polyhedra. Experimental structural data were compared with results from density functional theory (DFT) calculations employing a hybrid Hamiltonian. Valence region X-ray photoelectron and K-shell X-ray emission and absorption spectra of the LnInO_3_ compounds were simulated with the aid of the DFT calculations. Photoionization of lanthanide 4f orbitals gives rise to a complex final-state multiplet structure in the valence region for the 4f^*n*^ compounds PrInO_3_, NdInO_3_, and SmInO_3_, and the overall photoemission spectral profiles were shown to be a superposition of final-state 4f^*n*–1^ terms onto the cross-section weighted partial densities of states from the other orbitals. The occupied 4f states are stabilized in moving across the series Pr–Nd–Sm. Band gaps were measured using diffuse reflectance spectroscopy. These results demonstrated that the band gap of LaInO_3_ is 4.32 eV, in agreement with DFT calculations. This is significantly larger than a band gap of 2.2 eV first proposed in 1967 and based on the idea that In 4d states lie above the top of the O 2p valence band. However, both DFT and X-ray spectroscopy show that In 4d is a shallow core level located well below the bottom of the valence band. Band gaps greater than 4 eV were observed for NdInO_3_ and SmInO_3_, but a lower gap of 3.6 eV for PrInO_3_ was shown to arise from the occupied Pr 4f states lying above the main O 2p valence band.

## 1 Introduction

There is a growing interest in lanthanide indium perovskites LnInO_3_ related to their applications as phosphor materials,^1−3^ oxygen ion conductors,^4−9^ and photocatalysts.^10^ An all-perovskite field-effect transistor using LaInO_3_ as the gate material has also been reported.^11^ Most recently, it has been shown that a 2-dimensional electron gas may develop at the LaInO_3_/BaSnO_3_ interface,^12−16^ similar to that found for LaAlO_3_/SrTiO_3_.^17^ By analogy with BaSnO_3_, n-type doped LaInO_3_ also has potential as a transparent conducting oxide; donor doping on La site (e.g., by Ce) could in principle lead to a material where the donor centers are separated spatially from the In atoms contributing to the conduction band, thus suppressing ionized impurity scattering.^18^

LaInO_3_ itself adopts a perovskite structure based on an array of corner-shared InO_6_ octahedra, with the larger La^3+^ ions surrounded by 8 octahedra in a 12-coordinate site. A reduction in symmetry from the cubic perovskite structure to give an orthorhombic phase belonging to the *Pnma* space group arises from tilting of the InO_6_ octahedra to accommodate the deviation in the tolerance *t* factor defined by *t* = (*r*_La_ + *r*_O_)/√2(*r*_In_ + *r*_O_) from its ideal value of 1—here *r*_E_ refers to ionic radii of the elements. Based on the ionic radii tabulated by Shannon for 6-coordinate In^3+^, 12-coordinate Ln^3+^, and 2-coordinate O^2–^,^19^ the tolerance factor decreases across the early lanthanide from the value of 0.891 for LaInO_3_ and reaches 0.852 for SmInO_3_. This is near limit of about 0.78 for the range of stability for a perovskite phase and the ternaries EuInO_3_–HoInO_3_ adopt an alternative hexagonal *P*6_3_*cm* structure.^20,21^ The later lanthanide indates ErInO_3_–YbInO_3_ have been shown to belong to the cubic space group *Ia*3̅, with a structure related to that of In_2_O_3_.^20^ It must be assumed that LuInO_3_ has the same structure.

Our own interest in the LnInO_3_ perovskites arose from work on the growth of indium oxide thin films. Indium oxide doped with tin is a prototypical transparent conducting oxide with widespread applications as a transparent electrode in display devices, especially liquid crystal displays and many designs of solar cells. Recently, it has been possible to grow epitaxial thin films of In_2_O_3_ on a cubic Y-stabilized ZrO_2_ substrate due to the fact that 2*a* for Y–ZrO_2_ is tolerably close to *a* for In_2_O_3_, where *a* are the lattice parameters. However, a small mismatch of 1.7% places In_2_O_3_ epilayers under tensile strain, introducing dislocations or resulting in the breakup of thicker thin films.^22−27^ It was hypothesized that alloying In_2_O_3_ with an oxide of the early lanthanides (where the cation radius is bigger than that of In^3+^) would increase the lattice parameter in a solid solution, thus reducing epitaxial strain and allowing thicker films to be grown as single crystals. However, all attempts to prepare a doped phase In_2–*x*_La_*x*_O_3_ led to the appearance of orthorhombic lanthanum indium oxide LaInO_3_. This experience was repeated with Pr, Nd, and Sm as dopants.

The magnitude and nature of the band gap in LaInO_3_ is a matter of ongoing interest and controversy. In 1967, Rogers et al. suggested that LaInO_3_ has an electronic band gap of 2.2 eV separating states of dominant In 4d character at the top of the valence band from a conduction band formed from In 5s states.^28^ However, placement of In 4d levels above the O 2p valence band is at variance with an extensive body of experimental and theoretical work, which shows that in In_2_O_3_, the In 4d electrons occupy a shallow core level lying well below the bottom of the O 2p-dominated valence band. Hybridization between O 2p and In 4d states is rather weak, with only a small In 4d contribution to the states appearing at the top of the valence band.^29−34^ Nonetheless, the schematic band structure introduced by Rogers et al.^28^ was invoked recently to account for the optical properties of LaInO_3_ single crystals, in particular weak absorption beginning at about 2 eV.^35^ On the other hand, a much larger band gap of around 5 eV has been adopted in the literature dealing with LaInO_3_/BaSnO_3_ heterojunctions.^11−13,15,16^ Little is known about the electronic properties and band gaps of other lanthanide indium perovskites (PrInO_3_, NdInO_3_, and SmInO_3_), although it has been suggested recently that NdInO_3_ is a half-metallic ferromagnet.^36,37^ This in turn points to an unusual scenario where the 4f electrons are involved in itinerant electron ferromagnetism.

Prompted by the earlier interest in these materials, we present here a study of the properties of the four stable rare-earth indium perovskites using both experimental and computational approaches. It was first necessary to benchmark structural properties derived from density functional theory (DFT) calculations against structures derived from powder X-ray diffraction (XRD). Excellent agreement was achieved. Next, electronic structures were investigated using valence level X-ray photoelectron spectroscopy (XPS), along with X-ray emission and absorption spectroscopies (XES and XAS respectively), which fingerprint the O 2p contribution to the filled and empty states. Finally, band gaps were determined by diffuse reflectance optical spectroscopy, leading to the conclusion that the fundamental direct band gap of LaInO_3_ is greater than 4 eV and that absorption at much lower energies must be associated with defect states. The electronic structures emerging from the experimental work are shown to be in excellent agreement with results derived from DFT calculations using a hybrid Hamiltonian.

## 2 Experimental Section

Samples of LnInO_3_ (Ln = La, Pr, Nd, Sm) were prepared by high-temperature reaction between In_2_O_3_ (Sigma-Aldrich 99.999%) and either La_2_O_3_, Pr_6_O_11_, Nd_2_O_3_, or Sm_2_O_3_ (Sigma-Aldrich 99.99%). Precursor powders were ground together in an agate mortar in stoichiometric quantities and then pelletized between tungsten carbide dies under 5 tonnes for 5 min. The resulting ceramic pellets were fired at 1200 °C for 48 h and cooled to 600 °C over a period of 24 h. A further sample of LaInO_3_ was prepared by quenching rapidly to room temperature by removal from the hot furnace. The pellets were reground prior to measurement of θ–2θ powder XRD patterns in a PANalytical X’Pert diffractometer incorporating a monochromated Cu Kα source, but X-ray spectroscopic experiments were performed on the ceramic pellets. Preliminary XRD measurements showed that an orthorhombic perovskite phase had been prepared in each case. To confirm that the range of orthorhombic LnInO_3_ perovskites spans only from La to Sm, a synthesis of EuInO_3_ was conducted under the same firing conditions. The hexagonal phase of EuInO_3_ described by Pistorius^21^ was obtained. Attempts to prepare CeInO_3_ led to evaporation of In_2_O_3_, leaving CeO_2_ as a single phase. Powder patterns for the orthorhombic perovskites were refined using the General Structural Analysis System (GSAS) software package with the EXPGUI interface.

XPS of LaInO_3_ and PrInO_3_ were measured in a Scienta ESCA 300 spectrometer housed in Daresbury Laboratory UK. This incorporates a rotating anode Al Kα (*h*ν = 1486.6 eV) X-ray source, a 7 crystal X-ray monochromator, and a 300 mm mean radius spherical sector electron energy analyzer with parallel electron detection using channel plates, a scintillation screen, and a camera. The X-ray source was run with 200 mA emission current and 14 kV anode bias, while the analyzer operated at 150 eV pass energy. Gaussian convolution of the analyzer resolution with a linewidth of 260 meV for the X-ray source gives an effective instrument resolution of 400 meV. XPS of NdInO_3_ and SmInO_3_ were recorded at Cardiff University using a Kratos Axis Ultra spectrometer incorporating a fine focus monochromatic Al Kα X-ray source, a 165 mm mean radius spherical sector analyzer, and a delay line detector. The X-ray source was run with 10 mA emission current and 15 kV anode voltage. The analyzer pass energy was set at 40 eV, giving an overall energy resolution of 500 meV. Valence band and In 4d shallow core-level spectra are presented in the main body of the paper; Ln 3d core-level spectra are presented as Supporting Information.

XES and XAS experiments were conducted on beamline 7.0.1 at the Advanced Light Source Synchrotron, Lawrence Berkeley National Laboratory, USA. The beamline is based on a 20-period undulator and is equipped with a spherical grating monochromator to give a very intense source of soft X-rays.^38^ For X-ray absorption, both total electron yield (TEY) and total fluorescence yield (TFY) spectra were measured for the LnInO_3_ samples, although only TFY results are presented here. TFY is a photon-in photon-out process and is therefore less surface sensitive than the TEY. The energy resolution at the O K-edge was set to 200 meV. A reference current from a clean gold mesh, which was placed in the photon beam, was used to normalize the spectra. The XAS photon energy scale was calibrated using the O K-edge and Ti L-edge absorption peaks of rutile TiO_2_. Emission spectra were recorded using a Nordgren-type grazing-incidence spherical grating spectrometer with a resolution of 100 meV.^39^ The energy scale was calibrated relative to Zn Lα_1,2_ and Lβ_1_ emission lines of Zn metal.

Diffuse reflectance spectra in the wavelength range between 250 nm and 800 nm were measured using a PerkinElmer LAMBDA 750S ultraviolet/visible spectrometer incorporating a 60 mm integrating sphere. Measurements were performed both on ceramic pellets and ground-up pellets lightly pressed against KBr in a pellet press.

## 3 Computational Methodology

All calculations were performed using periodic DFT as implemented in the Vienna Ab Initio Simulation Package (VASP) in which a plane-wave basis set describes the valence electronic states.^40−43^ The Perdew–Burke–Ernzerhof (PBE)^44^ gradient-corrected functional was used to treat the exchange and correlation. The projector-augmented wave method^45^ was used to describe the interactions between the core and the valence electrons. In order to maximize computational efficiency, structural relaxations were carried out using Pr, Nd, and Sm pseudopotentials where f states were included in the core, while all electronic structure calculations explicitly included the Ln f states in the valence set. This resulted in an excellent reproduction of the experimental structural parameters without sacrificing the electronic properties such as the band gap and composition of the valence band. To counteract the self-interaction error and the band gap errors inherent to standard DFT functionals such as the PBE functional, higher levels of theory must be used. In this study, we have used the screened hybrid density functional developed by Heyd, Scuseria, and Ernzerhof (HSE06)^46^ as implemented in the VASP code. Difficulties in evaluating the Fock exchange in a real-space formalism are caused by the slow decay of the exchange interaction with distance. In the HSE06 hybrid functional approach, this problem is addressed by separating the description of the exchange interaction into long- and short-range parts, with a percentage (*a* = 25%) of exact nonlocal Fock exchange replacing the short-range (SR) PBE functional. A screening of ω = 0.11 bohr^–1^ is applied to partition the Coulomb potential into long-range (LR) and SR terms, which gives
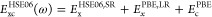
1
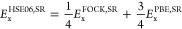
2

Fock and PBE exchanges are therefore only mixed in the SR part, with the LR exchange interactions being represented by the corresponding part of the range-separated PBE functional.

Structural optimizations of bulk LnInO_3_ were performed using HSE06 with a plane-wave energy cut-off of 600 eV and a Γ-centred *k*-point mesh of 4 × 4 × 3 allowing atomic positions, lattice vector and cell angle, and volume to relax. Calculations were deemed to be converged when the forces on all atoms were less than 0.01 eV Å^–1^. The calculations for all compounds were spin polarized such that spin-up and spin-down densities of states (DOS) were obtained separately.

Plotting of the DOS and band structures was carried out using the open-source Sumo package.^47^

## 4 Results and Discussion

### 4.1 Structure and InO_6_ Octahedral Tilt

**Figure 1:**
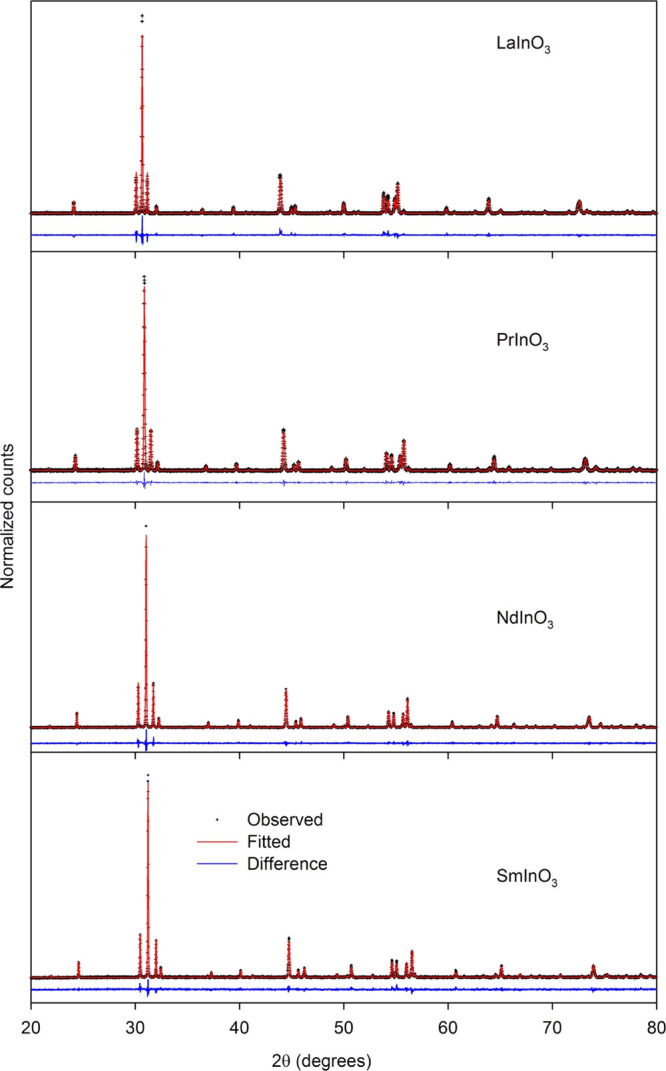
Measured X-ray powder diffraction patterns compared with results of Rietveld refinement for LaInO_3_, PrInO_3_, NdInO_3_, and SmInO_3_.

**Figure 2:**
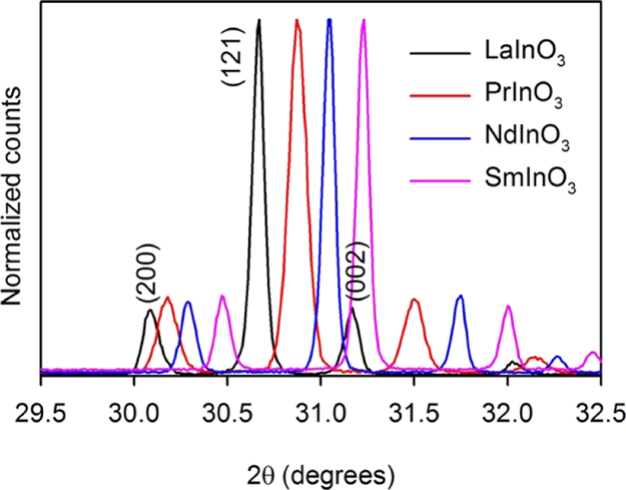
Expansion of diffraction patterns for 2θ values between 29.5 and 32.5° for LaInO_3_, PrInO_3_, NdInO_3_, and SmInO_3_ where the shift to low angle in the series La–Sm is apparent for the 200, 121, and 002 reflections.

Rietveld refinement of the structures for LaInO_3_, PrInO_3_, NdInO_3_, and SmInO_3_ in space group *Pnma* gave good fits to the experimental diffraction profiles, as shown in Figure 1. The structural parameters derived from the fits are given in Table 1, where they are compared with results from the DFT calculations. In broad terms, DFT reproduces all major trends found in the experimental data, in particular the decrease in lattice parameters across the series. The decrease is obvious when comparing expanded regions of the diffraction profiles for the four compounds, as in Figure 2, where shifts to high angle are found in moving from La to Sm for 200, 121, and 002 reflections (the *d*-spacings for these reflections would be the same for a cubic perovskite). The lattice parameters are in good agreement with those recently tabulated by Shukla et al.,^20^ while the fractional coordinates for LaInO_3_ match those found in an earlier powder XRD study.^48^ Likewise, fractional coordinates for PrInO_3_ are close to those from a powder neutron diffraction study,^49^ although of course in the current X-ray study, uncertainties in oxygen positions are bigger than in the neutron study.

**Table 1:**
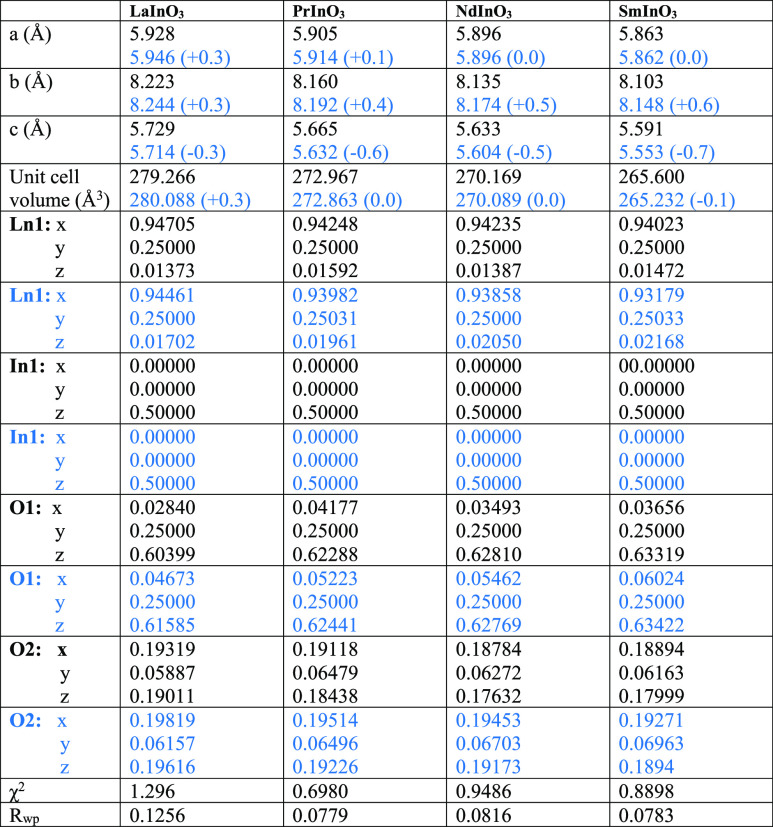
Lattice Parameters, Unit Cell Volumes, and Fractional Positional Coordinates for LnInO_3_ (Ln = La, Pr, Nd, Sm) Obtained from Rietveld Refinement Compared with Results from DFT Calculations in Blue Below^a^

a The percentage errors in calculated lattice parameters and cell volumes are given in parentheses.

**Figure 3:**
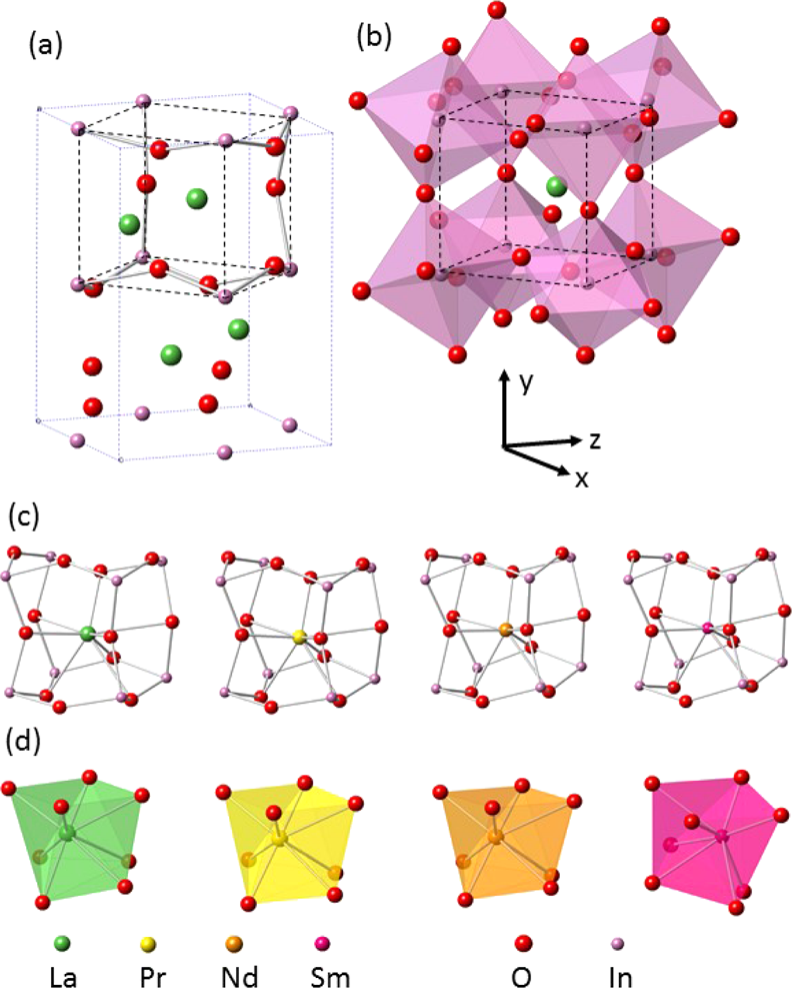
(a) Orthorhombic unit cell of a *Pmna* perovskite with a pseudo-cubic unit cell superimposed, defined by black dashed lines, (b) pseudo-cubic unit cell showing the 8 tilted InO_6_ octahedra surrounding each Ln atom, (c) experimental structures of the LnInO_3_ (Ln = La, Pr, Nd, Sm) perovskites represented in terms of a pseudo-cubic cell with corner-sharing InO_6_ octahedra, and (d) square antiprismatic coordination environment of the Ln ions in the perovskites, omitting 4 further O ions more than 3 Å distant from Ln^3+^.

The orthorhombic perovskite structure contains four formula units per cell and may be considered as a √2 × 2 × √2 superstructure of the unit cell of a cubic perovskite, with the √2 × √2 doubling in the *ac* (*xz*) plane, as shown in Figure 3. The lowering of symmetry arises from antiphase tilting of the adjacent octahedra, as discussed in detail by Glazer^50^ and Thomas.^51,52^ The tilting leads to elongation of 4 of the 12 equiv Ln–O bonds that would be found in the cubic phase accompanied by contraction of the remaining 8 bond lengths, leaving a Ln coordination environment with 8 oxygen ions in a distorted square anti-prismatic arrangement around each Ln ion. There are 4 more distant O neighbours. Overall, the changes in bond lengths lead to a decrease in the volume *V*_A_ within the structure occupied by the LnO_12_ polyhedra. The refined structures and Ln coordination environments are shown in panels (c,d) of Figure 3, while experimental and DFT Ln–O and In–O bond lengths are given as Supporting Information. The In–O octahedra remain almost regular both with the experimental and DFT results, and there is little change in the volume of the InO_6_ octahedra.

For an orthorhombic perovskite, it is possible to define three tilt angles θ_*x*′_, θ_*y*′_, and θ_*z*′_, where the θ are angles between In–In vectors in the pseudo-cubic unit cell (with axes *x*′, *y*′, and *z*′) and the component of the In–O vector involving the corner-shared O atoms projected onto the *x*′*z*′, *x*′*y*′, or *x*′*z*′ planes. Thomas has argued that for most practical purposes in orthorhombic, perovskites it is reasonable to ignore the differences between θ_*x*′_ and θ_*z*′_ and treat θ_*x*′,*z*′_ using an average value.^51,52^ It is then possible to define a consolidated tilt parameter ϕ given by
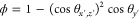
3

**Figure 4:**
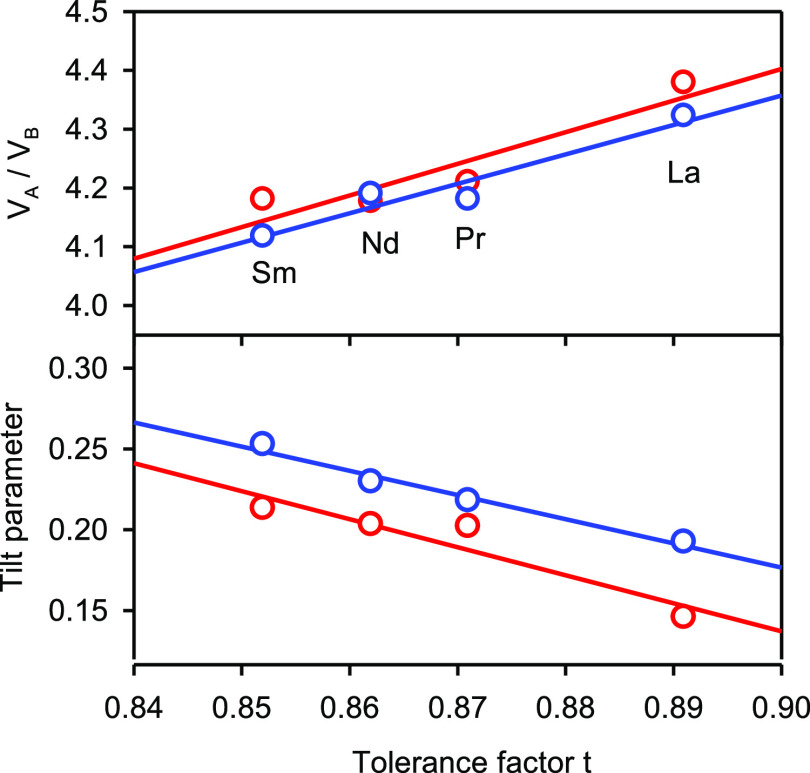
Upper: Ratio of LnO_12_ polyhedral volume (*V*_A_) to InO_6_ octahedral volume (*V*_B_) as a function of tolerance factor *t*. Lower: Thomas tilt parameter ϕ as a function of t. Experimental results in red; DFT results in blue.

These ideas are explored in Table 2, where values for cos θ_*x*′,*z*′_, cos θ_*y*′_, ϕ, and *V*_A_/*V*_B_ are given, while in Figure 4*V*_A_/*V*_B_ and ϕ are plotted as a function of the tolerance factor for both experimental Rietveld and DFT structures. In both cases, there is a general trend toward increasing tilt angles θ across the series from La to Sm, although the changes are not monotonic for the individual angles. However, the consolidated tilt parameter ϕ does increase in a monotonic fashion along the series La–Pr–Nd–Sm. As expected, the volume ratio *V*_A_/*V*_B_ decreases as the tolerance factor decreases.

**Table 2:**
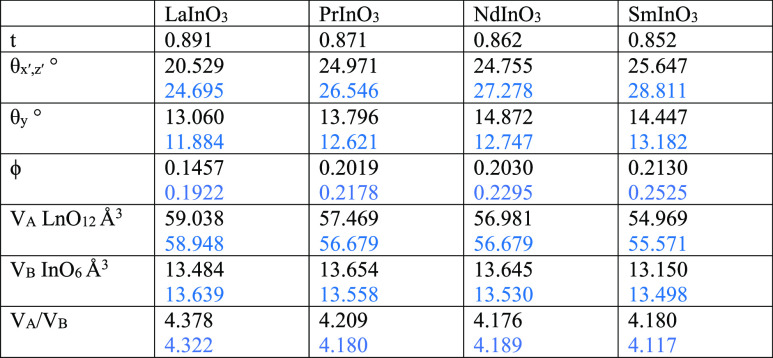
Tolerance Factors (*t*), Tilt Angles (θ_*x*′,*z*′_ and θ_*y*′_), Thomas Tilt Parameters (ϕ), and Polyhedral Volumes (*V*_A_ LaO_12_ and *V*_B_ InO_6_) for the LnInO_3_ Perovskites, Experimental Values Appear First with Values from DFT Calculations Shown in Blue Below

### 4.2 DFT Band Structures

**Figure 5:**
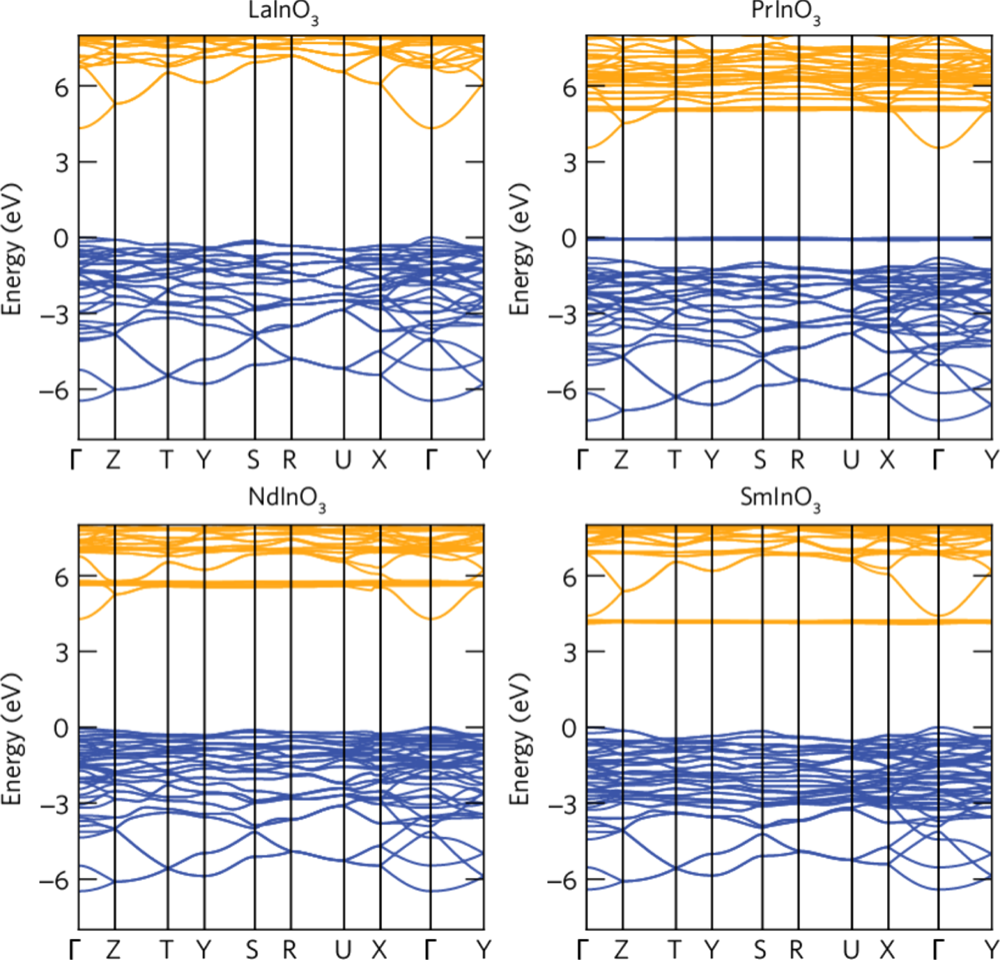
Band structure of LnInO_3_ perovskites (Ln = La, Pr, Nd, Sm). Occupied states are shown in blue, unoccupied states in yellow. The Fermi level coincides with the top of the filled states.

The band structure for the LnInO_3_ perovskites between −7 and +7 eV are shown in Figure 5. The overall width of the occupied valence bands (shown in blue) is about 6.4 eV in each case. There is no contribution from states of dominant In 4d atomic character in this region—the 4d bands lie much deeper (see below) and the bands shown in blue have their major contribution from O 2p orbitals. For LaInO_3_, the bottom of the lowest conduction band is separated from the top of the valence band by energy of 4.32 eV at the Γ point, giving a direct gap in the ultraviolet region of the electromagnetic spectrum.

It is notoriously difficult to calculate band gaps with DFT. However, it is becoming widely accepted that hybrid functionals such as that used here provide the simplest theoretical approach to reliable estimation of band gaps. Our current value is very close to that of 4.33 eV obtained by Krishnaswamy et al.^53^ using the same HSE06 functional as in our own calculations; minor differences in plane-wave cut offs and *k*-point sampling may account for the small discrepancy. However, our theoretical band gap is much bigger than the value of 2.55 eV proposed by Erkişi et al. on the basis of GGA + *U* calculations.^54^

The band structures for PrInO_3_, NdInO_3_, and SmInO_3_ each show the influence of new 4f states. For PrInO_3_, a non-dispersing band of occupied 4f states is found above the main O 2p valence bands, leading to a reduction of the bulk band gap, as will be discussed later. Weakly dispersing empty 4f bands about 2 eV above the conduction band minimum are also apparent. For NdInO_3_, the occupied 4f bands merge into the O 2p valence band states, but flat empty conduction band states again appear above the conduction band minimum. The present results are inconsistent with the idea that NdInO_3_ is a half-metallic ferromagnet, as suggested earlier.^36,37^ The occupied 4f bands are even more entangled with O 2p states for SmInO_3_. In addition, non-dispersing bands of 4f states now emerge below the bottom of the In 5s conduction band, leading to a small reduction of the calculated band gap. These results will be compared with experimental data later in the paper.

### 4.3 DFT Partial DOS

**Figure 6:**
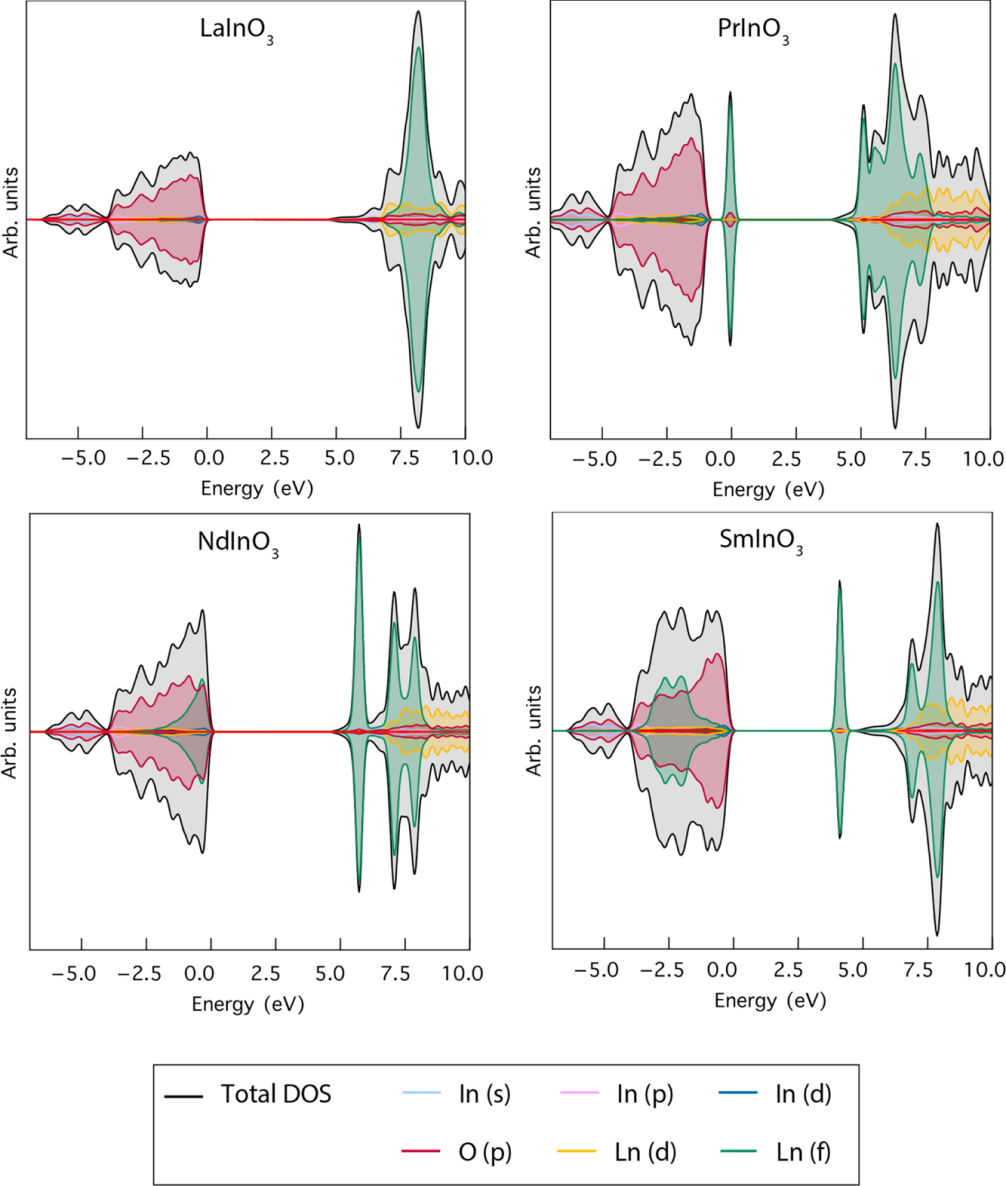
Spin-polarized PDOS for the LnInO_3_ perovskites (Ln = La, Pr, Nd, Sm).

Spin-polarized total and partial DOS (PDOS) for the perovskites are shown in Figure 6. As mentioned above, for LaInO_3_, the occupied states are of a dominant O 2p character, consistent with the idea that LaInO_3_ is basically a polar material containing La^3+^, In^3+^, and O^2–^ ions. Small covalent contributions from In 5s, 5p, and 4d as well as La 4f and 5d orbitals account for the small difference between the total and O 2p DOS and will be discussed further below. For LaInO_3_ itself, the 4f states are mainly confined to the conduction band, but in PrInO_3_ occupied Pr 4f states appear above the valence band maximum. There is also a broadening of the 4f contribution to the conduction bands below +10 eV, and emergence of a sharp (and presumably localized) component just above the conduction band minimum, as seen in the band structure. For NdInO_3_ and SmInO_3_, the occupied 4f contribution moves progressively downward into the O 2p valence band, while the lowest and sharp empty 4f structure also moves down in energy, appearing below the In 5s conduction band for SmInO_3_.

**Figure 7:**
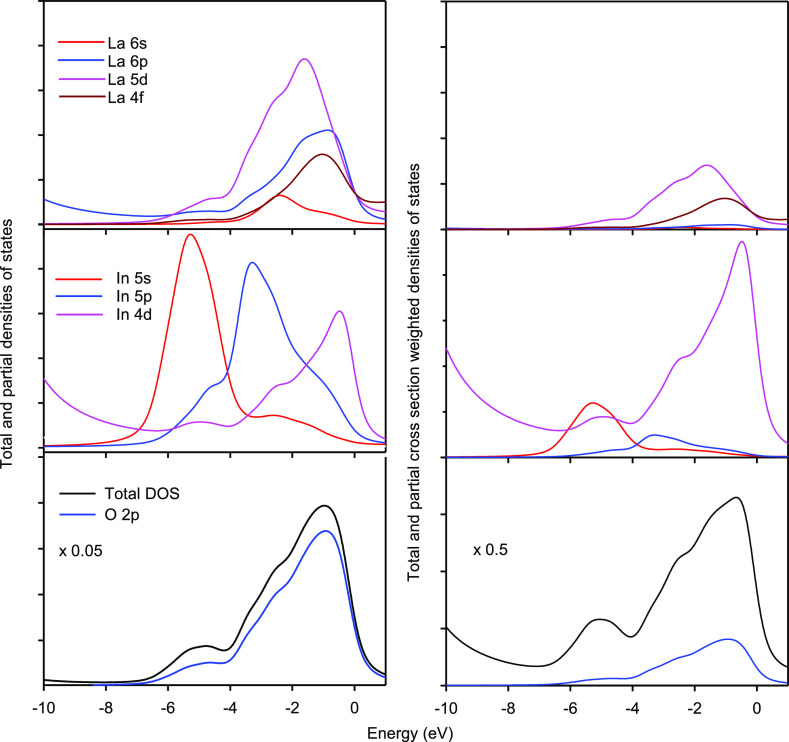
PDOS for LaInO_3_ (left) compared with CSWPDOS (right). The Ln and In partial contributions are presented on the same intensity scale, but the O 2p contributions and the overall DOS are scaled down by a factor of 20 before cross-section weighting and by a factor of 2 after this weighting. Cross-section weighting is seen to increase the importance of metal contributions.

The La 4f and 4d and In 5s, 5p, and 4d contributions are all presented on the same scale as the O 2p contribution in Figure 6, and it is difficult to identify the individual cationic contributions. This is remedied in Figure 7, where the cation contributions are expanded by a factor of 20 relative to O 2p. It is seen that In 4d contributes most to the top of the valence band, In 5p to the middle, and In 5s to the distinct peak at the bottom. This pattern differs from that for La states, where the most important La 5d contribution mimics the overall shape of the valence band.

To make comparison with experimental XPS, the individual PDOS need to be weighted with one electron ionization cross sections σ_*i*,*l*_ to give cross-section weighted PDOS (CSWPDOS), which are then summed to give a cross-section weighted total DOS (CSWTDOS)
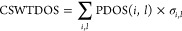
4where the summation extends over elements *i* and orbitals *l*. The cross sections relevant to the present study taken from the tabulation of Yeh and Lindau^55^ are given as Supporting Information Since the O 2p ionization cross section is less than that for the cation valence orbitals, the effect of cross-section weighting is to give increased emphasis to the contributions from the cation states, especially the In 4d and 5s states, as shown in the right hand panels of Figure 7.

A major difficulty arises in linking the calculated CSWTDOS to the experimental X-ray photoemission spectra for the perovskites with occupied 4f states. The 4f^n^ contribution to the spectra is determined not by the 4f CWPDOS but rather by the pattern of 4f^*n*–1^ final-state multiplets that are reached upon ionizing a localized 4f^*n*^ configuration. The energies of the multiplets are determined by interelectron repulsion and spin–orbit coupling, while the probability of reaching a given multiplet is determined by coefficients of fractional parentage. These coefficients show how the 4f^*n*^ initial state can be expanded using 4f^*n*–1^ final state wave functions coupled to f^1^. More specifically, in the *LS* limit, the probability *P* of reaching a final state (*S*′*L*′*J*′) from an initial state (*SLJ*) for a 4f^*n*^ configuration can be written as^56,57^
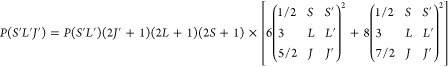
5where
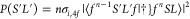
6

In eq 5, the 3 × 3 terms in brackets are Wigner 9j symbols, while in eq 6, the |⟨*f*^*n*–1^*S*′*L*′*f*|†}*f*^*n*^*SL*⟩| are the coefficients of fractional parentage. These ideas were extended by Gerken to deal with intermediate coupling, which allows excited states with different spin multiplicities to mix with the Russell–Saunders ground term, provided the states have the same *J* value—although this mixing is generally quite weak.^58^ Gerken did not treat the “trivial” case of 4f^2^, but this was analyzed by Beatham et al. using configuration interaction within a j–j basis.^59^ In the L–S limit, the ^3^H_4_ ground state of the 4f^2^ configuration gives rise to ^2^F_5/2_ and ^2^F_7/2_ final states, with an intensity ratio 1.714/0.286. In the j–j limit, only the ^2^F_5/2_ state is reached, while in intermediate coupling, the ratio lies between these the two limiting values (but toward the L–S end), with a value of 1.857/0.143.^59^

**Figure 8:**
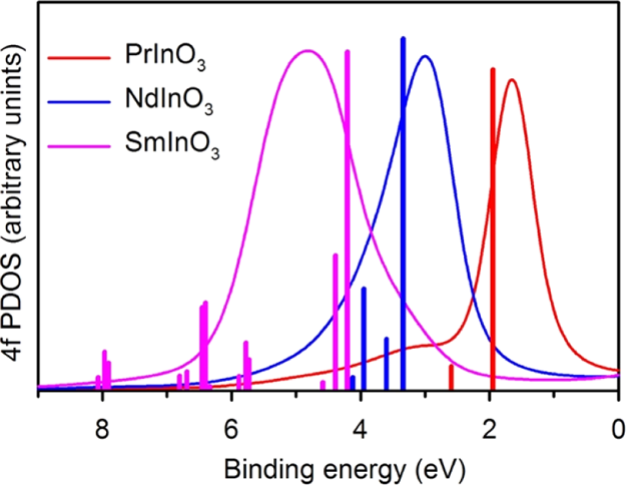
Comparison between the Ln 4f^*n*^ PDOS and the 4f^*n*–1^ multiplets for PrInO_3_, NdInO_3,_ and SmInO_3_.

Figure 8 compares the 4f^*n*^ PDOS for PrInO_3_, NdInO_3_, and SmInO_3_ with the pattern of 4f^*n*–1^ multiplets discussed above. The latter have been shifted to coincide with obvious features in the experimental spectra (see below). Although there seems to be an acceptable correspondence between the two approaches for PrInO_3_ and NdInO_3_, this is probably a matter of accident. For SmInO_3_, the two approaches give very different results.

### 4.4 Valence Region XPS and O K Shell XES and XAS

**Figure 9:**
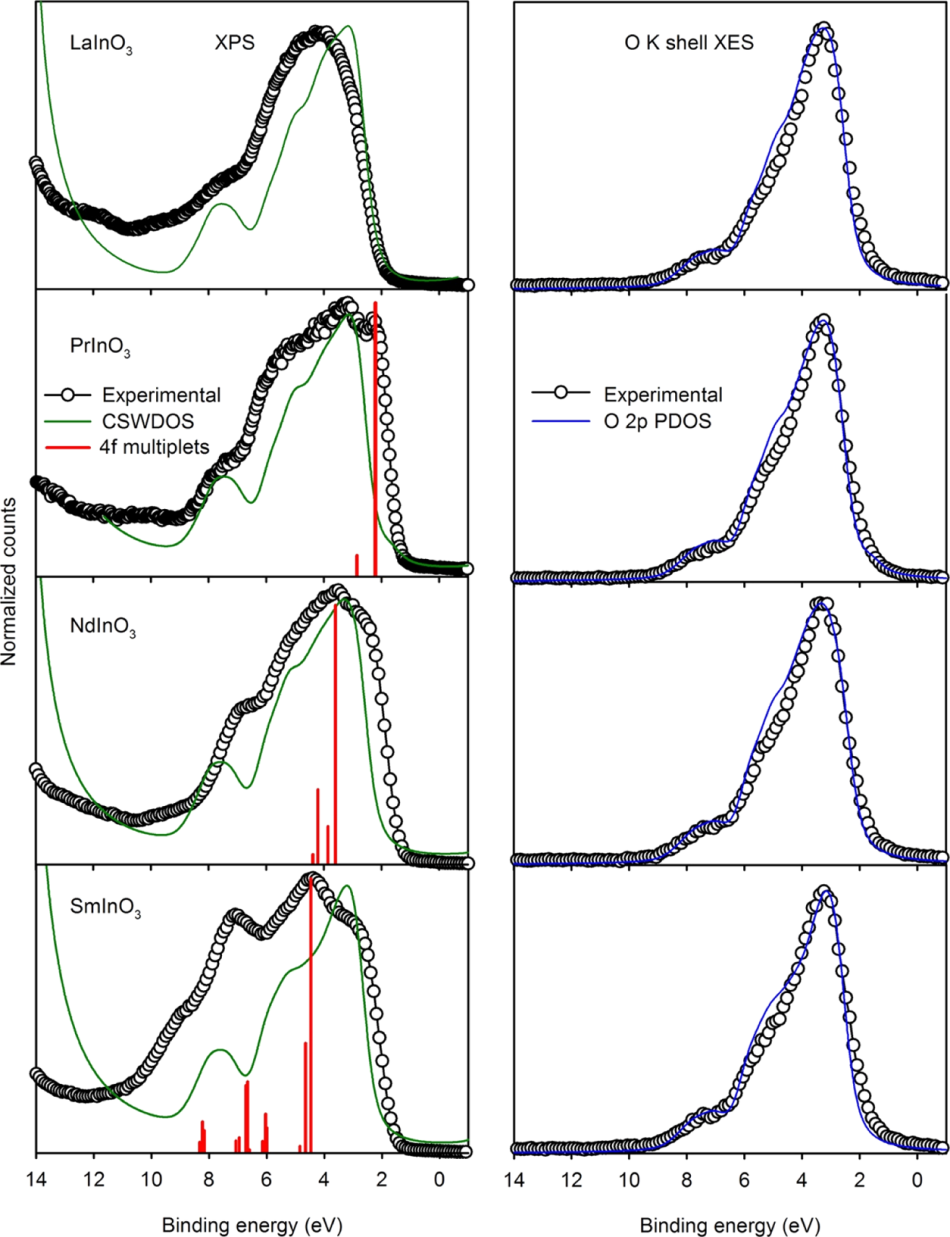
Valence band XPS and O K shell XES of LaInO_3_, PrInO_3_, NdInO_3_, and SmInO_3_. The experimental XPS data are compared with the CSWPDOS, excluding the Ln 4f contributions. The positions of 4f final-state multiplets calculated by Gerken are shown by vertical bars—the energies have been shifted rigidly to coincide with the 4f structure in the experimental spectra. The XES data have been shifted to coincide with the XPS data and are compared with the O 2p PDOS.

Figure 9 shows X-ray photoemission spectra and O K shell XES for the four perovskites. The photoemission spectra are compared with summations over CSWPDOS omitting the Ln 4f contribution as discussed above. Instead, the 4f^*n*–1^ final states are introduced as bars with the intensities and relative positions for the final-state multiplets given by Beatham et al. (PrInO_3_)^59^ and Gerken (NdInO_3_ and SmInO_3_).^58^ The multiplet positions have been shifted rigidly to coincide with obvious features in the experimental spectra. Overall, this hybrid approach reproduces the major features of the experimental spectra quite well, and it is possible to identify the 4f^*n*–1^ final states in the experimental spectra, even though they are superimposed on the remaining CSWDOS. Comparison between Figures 8 and 9 shows that for SmInO_3_ in particular the approach based solely on the CSWDOS derived from band theory does not match experimental spectra.

**Figure 10:**
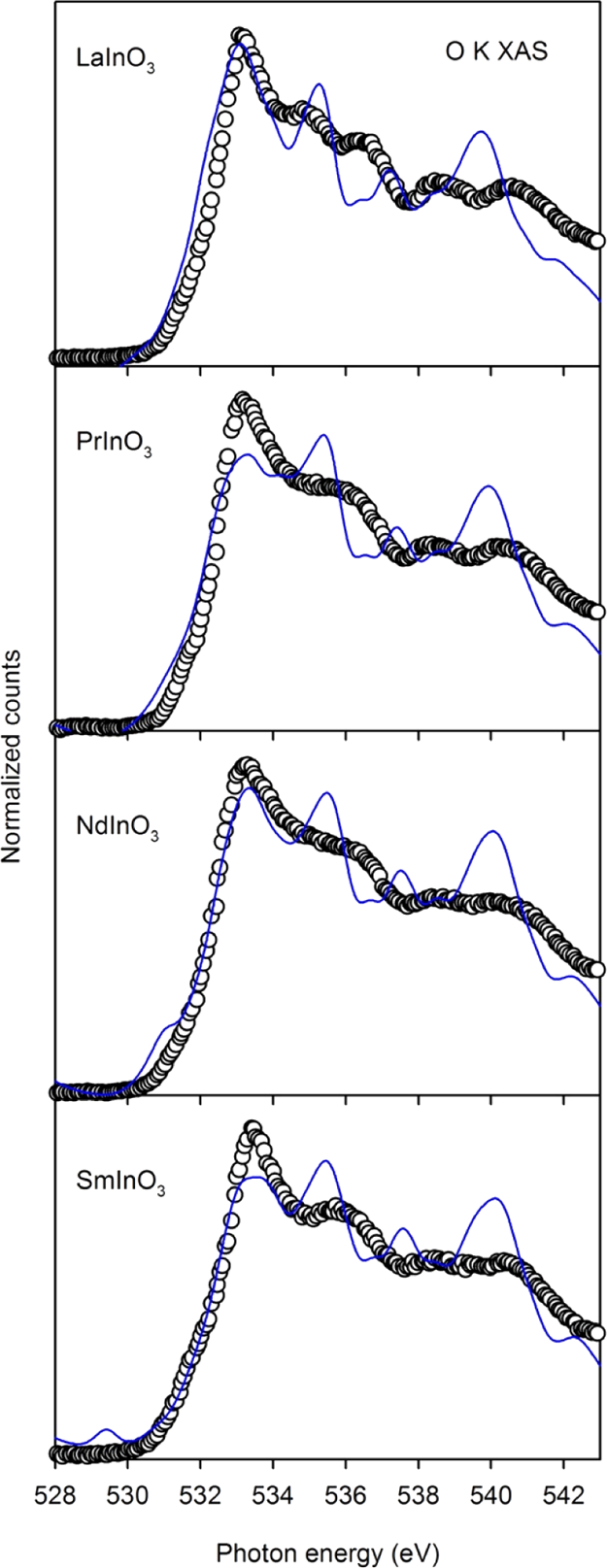
TFY XAS (open circles) compared with the unoccupied O 2p PDOS for LaInO_3_, PrInO_3_, NdInO_3_, and SmInO_3_. The PDOS curves have been shifted to coincide with the experimental data by energy that takes account of the initial state O 1s energy.

As shown in the right hand panels of Figure 9, O K shell X-ray emission for all four perovskites are very similar and as expected simply reflect the occupied O 2p PDOS: the decay of the O 1s core hole is governed by a strict dipole selection rule, which only allows transitions from filled O 2p states. Similarly, O K shell X-ray absorption probes the empty O 2p PDOS, and the absorption spectra shown in Figure 10 are all very similar. These findings are a consequence of the fact that the hybridization between Ln 4f states and O 2p states is exceedingly weak, so the O 2p structure remains essentially unchanged as the 4f orbital occupancy changes. We return to a discussion of the separation between X-ray absorption and emission edges in the penultimate section of the paper.

### 4.5 In 4d Level

**Figure 11:**
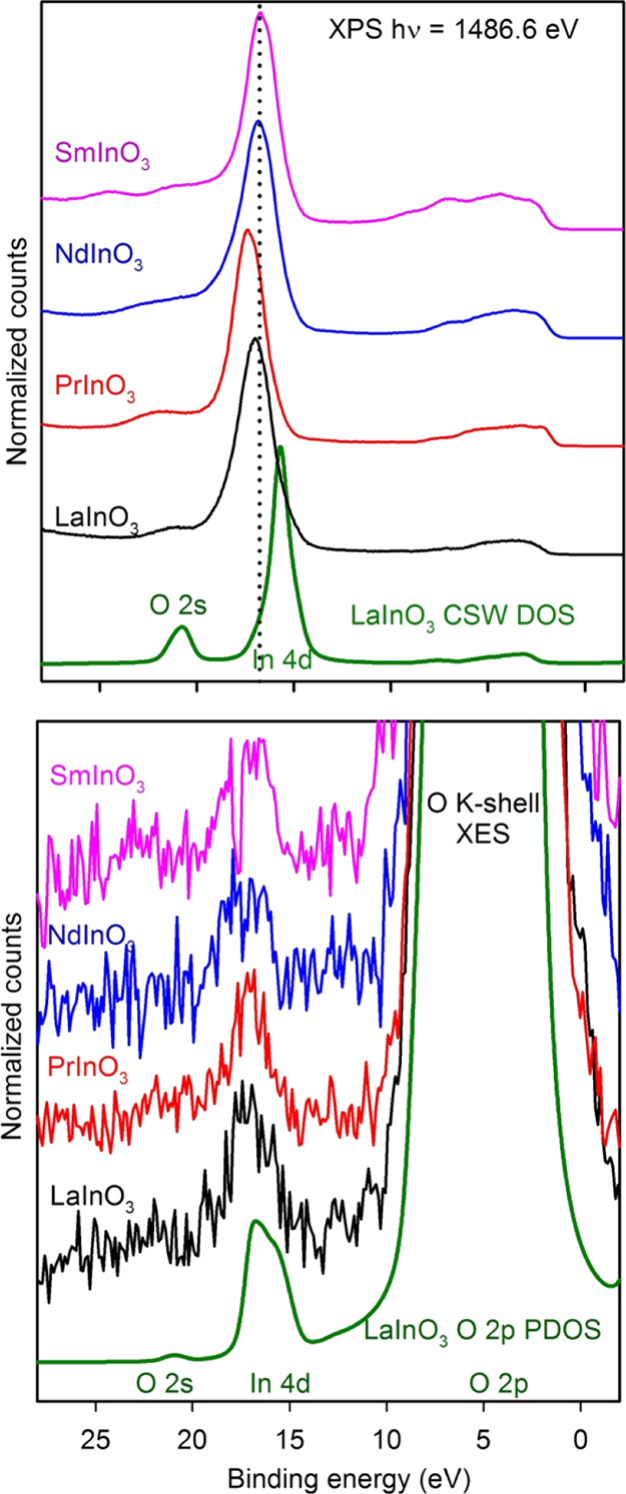
Upper panels: In 4d XPS of LaInO_3_, PrInO_3_, NdInO_3_, and SmInO_3_ compared with the CSWPDOS for the La compound. The In 4d level is seen to be a shallow core state, sitting about 16.75 eV below the Fermi level in each case, well below the minimum of the main valence bands. The calculated DFT CSWTDOS for LaInO_3_ is also shown. Lower panels: O K shell XES of the perovskites expanded to show the very weak hybridization between O 2p and In 4d shallow core states. The mixing is evident in the DFT O 2p PDOS shown for LaInO_3_.

Experimental valence band XPS spectra extended to include the In 4d level are shown in the top panel of Figure 11. Obviously, the In 4d level lies well below the bottom of the O 2p-dominated valence band and not above it, as suggested by Rogers et al.^28^ Binding energies of around 16.8 eV relative to the mid-gap level are in agreement with work on In_2_O_3_ and are reproduced quite well by the DFT calculations. Indium 4d is basically a shallow core level, although very weak mixing into O 2p states at the top of the valence band does occur, as has been discussed earlier. Likewise, O 2p mixes with In 4d, giving a small contribution to the O 2p PDOS at the binding energy of the In 4d level. This manifests itself by the appearance of a very weak structure in the O K shell XES at an energy corresponding to the In 4d level, as shown in the lower panel of Figure 11. From the intensity of the peak for LaInO_3_, it may be estimated that O 2p makes a 2.2% contribution to the In 4d core level. This mixing is comparable to that found for In_2_O_3_ but weaker than the mixing in for example CdO, where the Cd 4d level is shallower and a contribution of 4.3% may be estimated.^60,61^ CdO adopts a centrosymmetric rocksalt structure and mixing between ungerade O 2p and gerade Cd 4d states is not possible at the Γ point due to the differing parities. However, mixing away from Γ is possible leading to O 2p–Cd 4d antibonding states at the top of the valence band and an anomalously low indirect band gap.^62^ There is no hint from the bandstructure of comparable effects for LaInO_3_ and a band gap of around 2 eV could not be explained by hybridization creating strongly antibonding states at the top of the valence band.

### 4.6 Diffuse Reflectance Spectroscopy and Experimental Band Gaps

**Figure 12:**
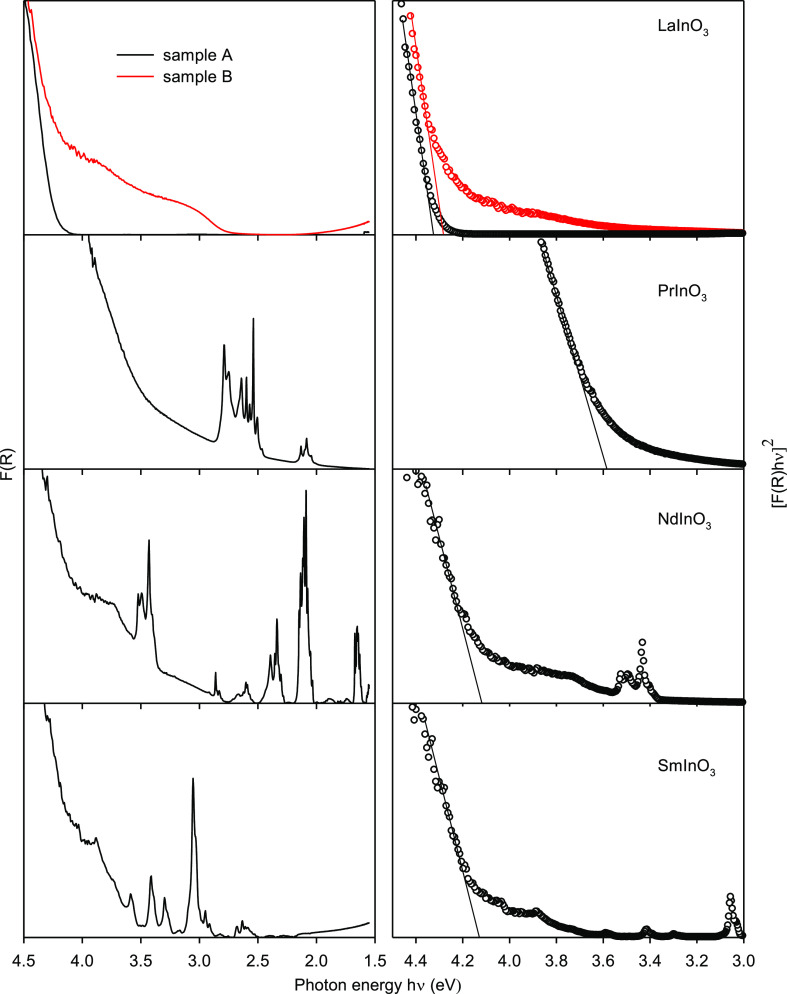
Left hand panels: diffuse reflectance spectra of LaInO_3_, PrInO_3_, NdInO_3_, and SmInO_3_ presented with the Kubelka–Munk function *F*(*R*) plotted against photon energy *h*ν. Right hand panels: Expansions of the band-edge region with [*F*(*R*)*h*ν]^2^ plotted against *h*ν.

Diffuse reflectance spectra with the Kubelka–Munk function *F*(*R*) (which is proportional to the absorption coefficient) plotted against photon energy are shown in the left hand panels of Figure 12. The onset of strong absorption for the slow-cooled sample of LaInO_3_ lies above 4.0 eV in energy. However, for the quenched sample, a weak double-structured tail extends down to about 2.5 eV. Comparison with the DFT calculations suggests that the weak visible region absorption is not related to the bulk band gap: in particular, the calculations rule out the possibility that the weak absorption is associated with an indirect band gap. Instead, the weak absorption must be associated with defect states of some sort, probably arising from oxygen vacancies present under conditions of high-temperature synthesis and “frozen” into the sample prepared by rapid cooling. One possibility is that the two electrons associated with an O vacancy are trapped on an adjacent indium ion to give an In(I) “lone pair” state. States of this sort have recently been characterized on vacuum-annealed In_2_O_3_(111) surfaces and lie above the valence band maximum in photoelectron spectra.^63^ Note that the presence of In(I) adatoms on the surface of In_2_O_3_(111) does not produce a chemically shifted component in the In 4d and 3d core lines. Further experimental and computational work on the defect chemistry of LaInO_3_ is needed to explore the tentative suggestion that low-energy absorption in optical spectra is associated with In(I) lone pair states. In particular, it needs to be established experimentally if oxygen vacancies are indeed present in quenched samples, if the presence of these vacancies leads to lone pair states in the bulk of the perovskite, and finally if “bulk” lone pair states lie at similar energy to those found on In_2_O_3_ surfaces.

Additional evidence for a band gap in excess of 2 eV comes from the separation of the valence band and conduction band edges in XES/XAS. The two superimposed spectra are presented as Supporting Information. The “raw” separation between the edges is about 2.2 eV, but the absorption spectra need to be shifted to higher energy to deal with the influence of the core hole potential. For oxide materials, shifts of the order of 1–2 eV have been suggested,^64,65^ leading to a band gap in the range 3.2–4.2 eV. It remains to be explained why the band gap for LaInO_3_ found in the current work on ceramic samples is significantly lower than the value of 5 eV favored in the community working on thin-film material.^11−13,15,16^ One possibility is that the lowest energy interband transitions are dipole forbidden or have weak dipole intensity, as is the case for In_2_O_3_^31^ and are therefore difficult to observe in absorption spectra of thin films.

The optical spectra of PrInO_3_, NdInO_3_, and SmInO_3_ are considerably more complicated than that of LaInO_3_, with sharp peaks associated with localized 4f → 4f transitions to the low energy of the interband onsets. It is beyond the scope of the current paper to discuss assignment of this structure in detail, but in general terms, the 4f → 4f bands are very similar to those for the simple Ln_2_O_3_ oxides or more complex doped materials such as the Bi_0.5_Ln_0.5_VO_4_ vanadates.^66^

The band structure calculations show that the band gaps for all four perovskites are either direct (LaInO_3_ and NdInO_3_) or that the energy difference between a lower energy indirect gap and the direct gap is so small (<0.01 eV) as to be negligible (PrInO_3_ and SmInO_3_). It is therefore appropriate to estimate the position of the interband absorption onset by plotting (*F*(*R*)*h*ν)^2^ against photon energy *h*ν. Plots of this sort are shown in the right-hand panels of Figure 12. Obviously, the presence of the localized excitations introduces some difficulties in defining the positions of the onsets of interband excitations, although moving from *F*(*R*) to (*F*(*R*)*h*ν)^2^ diminishes the intensity of the 4f → 4f bands relative to that of the interband excitation at higher energy. The best estimates for the experimental band gaps are given in Table 3, along with the values derived from the DFT calculations.

**Table 3:**
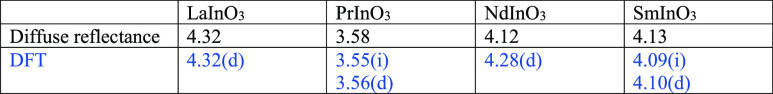
Experimental Band gaps for the LnInO_3_ Perovskites Compared with Values from DFT Calculations^a^

a All values are in eV.

The experimental gap for PrInO_3_ is lower than for LaInO_3_ and in good agreement with DFT; as noted previously, the smaller gap arises from occupied Pr 4f states above the main O 2p valence band. The experimental gaps for NdInO_3_ and SmInO_3_ are, respectively, 4.13 and 4.12 eV, intermediate between the values for LaInO_3_ and PrInO_3_. The computed values for the band gaps are 4.28 and 4.09 eV. These are tolerably close to the experimental values, but the reduction in band gap by 0.19 eV between NdInO_3_ and SmInO_3_ found in the computations is not found experimentally. The smaller gap for SmInO_3_ in the computations arises from a weakly dispersing band of empty 4f states just below the In 5s conduction band minimum, but as we have seen, DFT has difficulties in dealing with quasi-localized 4f states.

The direct band gap of 4.3 eV for LaInO_3_ found in the current study is much greater than the experimental value of just over 3 eV now generally accepted for the closely related perovskite BaSnO_3_, where the lowest energy gap is indirect.^12,16,67−70^ This contrasts with the situation for the binary oxides of In and Sn, where the direct but dipole forbidden band gap of In_2_O_3_^31,71^ (2.9 eV for the bixbyite phase) is less than that of SnO_2_ (3.6 eV).^72,73^ To gain some insight into the reasons for these differences, it is helpful to revisit ideas first introduced by Goodenough.^74^ In ABO_3_ perovskite materials, band gaps and bandwidths are determined by indirect B–O–B interactions. Two factors influence the strength of these interactions. First, B–O–B mixing is strongest when the B–O–B bonds are linear. Second, the A ions in a perovskite “compete” with the B ions in their interaction with oxygen orbitals and the more “acidic” A, the weaker the B–O–B interactions.^74−76^ Since La^3+^ is more acidic than Ba^2+^ (because its empty acceptor orbitals are lower in energy), both these factors favor stronger B–O–B interactions in BaSnO_3_ than LaInO_3_. These qualitative ideas are incorporated in a natural way in hybrid DFT calculations which give theoretical values for the indirect and direct gaps of BaSnO_3_ in excellent agreement with experimental results.^65,77,78^ The curvature of the lowest conduction band for LaInO_3_—which has pronounced In 5s character—corresponds to an effective mass ratio m*/*m*_0_ of about 0.5. This is higher than the values of 0.22 for In_2_O_3_^79,80^ and of 0.20–0.26 for BaSnO_3_.^53,65,77,78^ The enhanced effective mass in LaInO_3_ as compared with BaSnO_3_ is a further consequence of the reduced O-mediated B–B interactions in the latter compound.

## 5 Concluding Remarks

Orthorhombic lanthanide indium perovskites LaInO_3_, PrInO_3_, NdInO_3_, and SmInO_3_ belonging to the space group *Pnma* were prepared by reaction between In_2_O_3_ and the appropriate lanthanide oxide at elevated temperatures. Experimental structural parameters for the perovskites derived from Rietveld refinement of powder XRD patterns were found to be in excellent agreement with results from DFT calculation employing a hybrid Hamiltonian. In particular, both approaches revealed increased tilting of the InO_6_ octahedra across the series La–Pr–Nd–Sm associated with a progressive decrease of the tolerance factor as the lanthanide ion decreases in size.

DFT shows that for LaInO_3_, a valence band of O 2p derived states is separated from the conduction band of In 5s states by a large band gap of 4.3 eV, a value confirmed by diffuse reflectance spectroscopy. The occupied In 4d and unoccupied La 4f states lie, respectively, well below the top of the valence band and well above the bottom of the conduction band. These assignments are supported by the excellent agreement between DFT orbital projections considered alongside XPS, XES, and XAS. Most notably, the differing orbital selectivities of XPS and XES/XAS were used to confirm the DFT calculations and to demonstrate that the In 4d levels are located deep below the valence band minimum. Thus, absorption at around 2.2 eV reported previously cannot be associated with transitions from In 4d states into the conduction band. It is tentatively suggested that In(I) lone pair states resulting from oxygen vacancies are responsible for the low energy absorption onset, but further experimental and theoretical work is needed to investigate this hypothesis.

Moving across the lanthanide series, the smaller band gap of 3.8 eV for PrInO_3_ observed in diffuse reflectance spectroscopy was shown to arise from occupied Pr 4f states lying just above the valence band O 2p states, whereas for NdInO_3_ and SmInO_3_, the localized occupied 4f states are embedded within the valence band and energy gaps similar to that in LaInO_3_ are found. There is no evidence that NdInO_3_ is a half-metallic ferromagnet, as has been suggested in the literature.^36,37^

The value of the band gap for LaInO_3_ found in the current work will be important in the future development of ideas about band alignment at LaInO_3_/BaSnO_3_ interfaces. The value of 5 eV for the band gap of LaInO_3_ favored by those working in this area appears to be too big, but further work is needed to explain the discrepancy with the findings presented here. Finally, we note that the evident stability of the perovskite phases studied in the current work probably excludes the possibility of tuning the lattice parameter of indium oxide by alloying with one of the early lanthanide oxides.

## Supporting Information Available

Experimental metal–oxygen bond lengths, one electron ionization cross sections, lanthanide 3d core levels, and estimation of band gap of LaInO_3_ from comparison of XES and XAS (PDF)
