# Supplementary material for: Experimental and Theoretical Study of the Electronic Structures of Lanthanide Indium Perovskites LnInO3
Source: J Phys Chem C Nanomater Interfaces. 2021 Mar 11;125(11):6387–400. doi: 10.1021/acs.jpcc.0c11592 (PMC8042864; doi:10.1021/acs.jpcc.0c11592)
Supplement: Supplementary file 1 — jp0c11592_si_001.pdf [file jp0c11592_si_001.pdf]

Supporting Information

Experimental and Theoretical Study of the Electronic Structures of Lanthanide Indium Perovskites $LnInO_3$

P. Hartley,[1] R.G. Egdell,[1] K.H.L. Zhang,[1,2] M. V. Hohmann,[1,3] L.F.J. Piper,[4,5]

D. J. Morgan,[6] D.O. Scanlon,[7-9] B.A.D. Williamson*,[10] A. Regoutz[1,7]

[1] Department of Chemistry, University of Oxford, Inorganic Chemistry Laboratory, South Parks Road, Oxford, OX1 3QR, UK.

[2] State Key Laboratory of Physical Chemistry of Solid Surfaces, College of Chemistry and Chemical Engineering, Xiamen University, Xiamen 361005, Peoples Republic of China.

[3] Institute of Materials Science, Surface Science Division, Technische Universität Darmstadt, Darmstadt 64287, Germany.

[4] WMG, The University of Warwick, Coventry, CV4 7AL, UK.

[5] Department of Applied Physics & Astronomy, Binghamton University State University of New York, USA.

[6] Cardiff Catalysis Institute, School of Chemistry, Cardiff University, Park Place, Cardiff CF10 3AT, UK.

[7] Department of Chemistry, University College London, 20 Gordon Street, London WC1H 0AJ, UK

[8] Thomas Young Centre, University College London, Gower Street, London WC1E 6BT, UK

[9] Diamond Light Source Ltd., Diamond House, Harwell Science and Innovation Campus, Didcot, Oxfordshire, OX11 0DE, UK

[10] Department of Materials Science and Engineering, Norwegian University of Science and Technology (NTNU), Trondheim 7491, Norway

*Corresponding author. Email: benjamin.williamson@ntnu.no

S1 Experimental metal-oxygen bond lengths

Experimental metal oxygen bond lengths are given in table S1.

Table S1 Experimental and DFT (in brackets) In-O and Ln-O bond lengths in the lanthanide indium perovskites.

	$LaInO_3$	$PrInO_3$	$NdInO_3$	$SmInO_3$
In-O<br>bond lengths<br>(Å)	2.147 (2.159)<br>2.147 (2.159)<br>2.167 (2.179)<br>2.167 (2.179)<br>2.175 (2.183)<br>2.175 (2.183)	2.167 (2.147)<br>2.167 (2.147)<br>2.170 (2.171)<br>2.170 (2.171)<br>2.180 (2.187)<br>2.180 (2.187)	2.153 (2.144)<br>2.153 (2.144)<br>2.168 (2.168)<br>2.168 (2.168)<br>2.193 (2.190)<br>2.193 (2.190)	2.134 (2.138)<br>2.134 (2.138)<br>2.136 (2.162)<br>2.136 (2.162)<br>2.164 (2.197)<br>2.164 (2.197)
Ln-O<br>bond lengths.<br>Primary co-ordination sphere (Å)	2.371 (2.371)<br>2.396 (2.395)<br>2.396 (2.395)<br>2.572 (2.485)<br>2.759 (2.692)<br>2.759 (2.692)<br>2.916 (2.967)<br>2.916 (2.967)	2.302 (2.323)<br>2.313 (2.352)<br>2.313 (2.352)<br>2.493 (2.431)<br>2.715 (2.641)<br>2.715 (2.649)<br>2.917 (2.950)<br>2.917 (2.965)	2.241 (2.305)<br>2.292 (2.332)<br>2.292 (2.332)<br>2.532 (2.411)<br>2.760 (2.628)<br>2.760 (2.629)<br>2.865 (2.954)<br>2.865 (2.960)	2.170 (2.269)<br>2.298 (2.297)<br>2.298 (2.297)<br>2.502 (2.373)<br>2.704 (2.598)<br>2.704 (2.599)<br>2.845 (2.954)<br>2.845 (2.969)
Ln-O<br>bond lengths.<br>Secondary co-ordination sphere (Å)	3.416 (3.475)<br>3.512 (3.660)<br>3.800 (3.804)<br>3.800 (3.804)	3.488 (3.471)<br>3.625 (3.711)<br>3.848 (3.831)<br>3.848 (3.838)	3.503 (3.471)<br>3.584 (3.726)<br>3.852 (3.844)<br>3.852 (3.845)	3.443 (3.477)<br>3.589 (3.753)<br>3.805 (3.856)<br>3.805 (3.860)

S2 One electron ionisation cross sections

One electron ionisation cross sections used in simulation of valence region photoemission spectra are given in table S2.[1]

Table S2 One electron photoionisation cross section for valence orbital in $LnInO_3$ compounds.

	**La**	**Pr**	**Nd**	**Sm**	**Gd**
**6s**	145	190	170	145	
**6p**	97	127	113	97	
**5d**	760	811*	837*	888*	940
**4f**	867#	1333	1575	2000	

	**In**	**Sn**	**Sb**
**5s**	500		
**5p**	237≠	385	533
**4d**	3100		
	**O**		
**2s**	950		
**2p**	60		

* Linearly interpolated cross section from La 5d and Gd 5d. #Linearly extrapolated check Pr 4f and Nd 4f. ≠ Linearly extrapolated cross section from Sn 5p and Sb 5p. All values are given in barns (1 barn = 10^{-28} m^2)

S3 Lanthanide 3d core levels

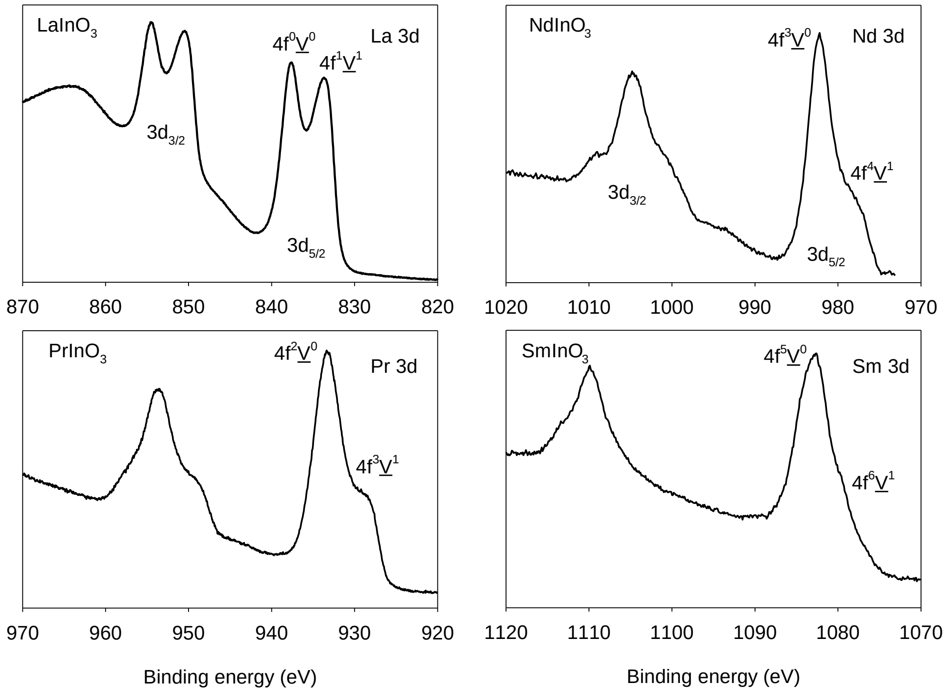

Fig. S3. Ln 3d core level photoelectron spectra for $LaInO_3$, $PrInO_3$, $NdInO_3$ and $SmInO_3$.

Ln 3d core level photoelectron spectra of the four lanthanide perovskites are shown in Fig. S3. Spin-orbit coupling leads to a splitting between 5/2 and 3/2 components that increases in moving across the lanthanide series from La to Sm. Each component for $LaInO_3$ is further split into two, with clearly separated peak maxima. For the other perovskites there are also two components for the 5/2 peak, but with a low binding energy component comprised of a shoulder rather than a distinct peak: the shoulder becomes progressively weaker in the series Pr-Nd-Sm. The 3/2 peaks are more complex for the $PrInO_3$, $NdInO_3$ and $SmInO_3$ with shoulders to both low and high binding energy of the main peak.

The origins of this splitting have been discussed extensively in the published literature in terms of configuration mixing in the initial state and final state screenings.[2-4] In outline, the high binding energy component of $3d_{5/2}$ for $LaInO_3$ arises from a final state corresponding to a $4f^0$ valence configuration, whereas the component at lower binding energy is associated with a

screened $4f^1$ in the final state, with a hole in the valence band. As we have seen in the main body of the paper the valence band is comprised mainly of O 2p states. These two configurations are identified as $4f^0\underline{V}^0$ and $4f^1\underline{V}^1$ in the figure where V is the valence band and the underscore indicates a hole. The $4f^1$ configuration is present to some extents in the initial state due to a small extent of La 4f−O 2p covalency, but the intensity is enhanced by final state charge-transfer screening from oxygen into the 4f level induced by the core hole potential. Similar assignments apply to the $3d_{5/2}$ peaks for $PrInO_3$, $NdInO_3$ and $SmInO_3$, with the high binding energy unscreened peak corresponding to the $4f^n$ electron configuration in the initial state and the low binding energy component to $4f^{n+1}\underline{V}^1$.

The intensity of the screened component decreases progressively in traversing the lanthanides and the peaks become broader. Both this broadening and the high binding energy shoulder found for the $3d_{3/2}$ peaks may be linked to exchange and orbital coupling between the 3d core hole and the open shell 4f configurations.[5]

S4 Estimation of band gap of $LaInO_3$ from comparison of XES and XAS

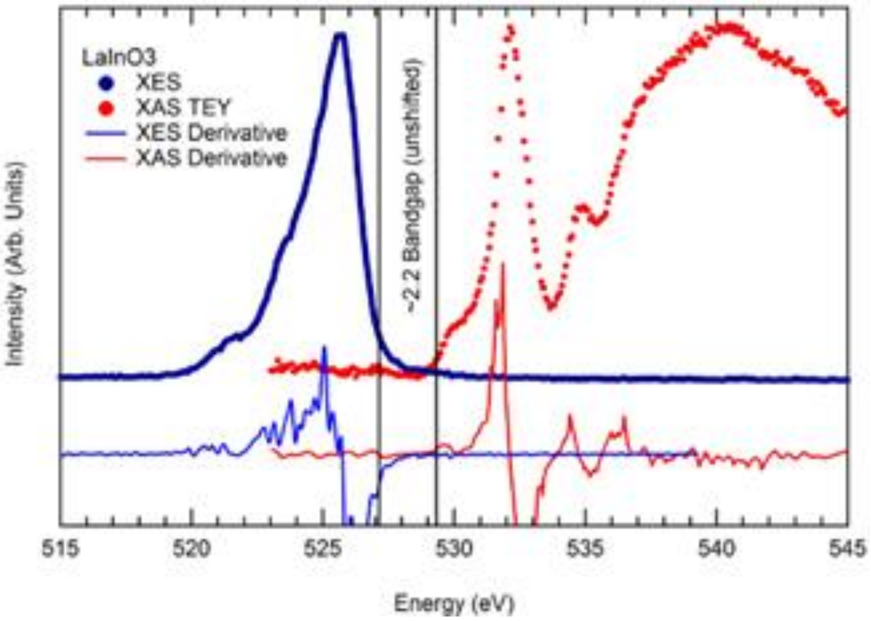

Fig. S4 XES (blue dots) and total electron yield (TEY) XAS (red dots) for $LaInO_3$ presented on the same energy scale. The derivates are shown below as solid lines.

XES and total electron yield XAS spectra presented on the same energy scale are shown in Fig. S4. The positions of the band edges may be defined in terms of inflexion points in the first derivative curves. This analysis reveals a 2.2 eV separation between the band edges. However to estimate the band gap from these data a correction must be applied to accounftfor the influence of the potential associated with the core hole on the absorption spectra. In previous work on oxide shifts of order 1 eV – 2 eV have been invoked,[6,7] and with these corrections we obtain estimates for the bnad gap of between 3.2 eV and 4.2 eV.

REFERENCES

1. Yeh, J. J.; Lindau, I., Atomic Subshell Photoionization Cross-Sections and Asymmetry Parameters - 1 Z 103. *At. Data Nucl. Data Tables* **1985,** *32*, 1-155.

2. Burroughs, P.; Hamnett, A.; Orchard, A. F.; Thornton, G., Satellite Structure in X-Ray Photoelectron Spectra of some Binary and Mixed Oxides of Lanthanum and Cerium. *Journal of the Chemical Society-Dalton Transactions* **1976**, 1686-1698.

3. Suzuki, C.; Kawai, J.; Takahashi, M.; Vlaicu, A. M.; Adachi, H.; Mukoyama, T., The Electronic Structure of Rare-Earth Oxides in the Creation of the Core Hole. *Chemical Physics* **2000,** *253*, 27-40.

4. Kotani, A.; Ogasawara, H., Theory of Core-Level Spectroscopy of Rare-Earth-Oxides. *Journal of Electron Spectroscopy and Related Phenomena* **1992,** *60*, 257-299.

5. Kotani, A.; Ogasawara, H., Interplay between Intra-Atomic Multiplet Coupling and Interatomic Hybridization in Core-Level Spectroscopy. *Journal of Electron Spectroscopy and Related Phenomena* **1997,** *86*, 65-72.

6. Sallis, S.; Scanlon, D. O.; Chae, S. C.; Quackenbush, N. F.; Fischer, D. A.; Woicik, J. C.; Guo, J. H.; Cheong, S. W.; Piper, L. F. J., La-doped BaSnO3-Degenerate perovskite transparent conducting oxide: Evidence from synchrotron x-ray spectroscopy. *Applied Physics Letters* **2013,** *103*, 042105.

7. On-site Interband Excitations in Resonant Inelastic X-ray Scattering from Cu_2O. Hu, J.P.; Payne, D.J.; Egdell, R.G.; Glans, P-A.; Learmonth, T.; Smith, K.E.; Guo, J.; Harrison, N.M. *Phys. Rev. B* **2008**, *77*, 155115
